# Glimepiride and Metformin Combinations in Diabetes Comorbidities and Complications: Real-World Evidence

**DOI:** 10.7759/cureus.10700

**Published:** 2020-09-28

**Authors:** Rakesh Kumar Sahay, Vinod Mittal, G Raja Gopal, Sunil Kota, Ghanshyam Goyal, Mahesh Abhyankar, Santosh Revenkar

**Affiliations:** 1 Department of Endocrinology, Osmania Medical College, Hyderabad, IND; 2 Centre for Diabetes & Metabolic Diseases, Delhi Heart & Lung Institute, New Delhi, IND; 3 Department of Endocrinology and Diabetes, Sriridhi Endocrinology & Diabetes Super Specialty Clinic, Kurnool, IND; 4 Department of Endocrinology, Endocare Hospital, Vijaywada, IND; 5 Department of Endocrinology, S K Diabetes Research and Education Centre, Kolkata, IND; 6 Scientific Services, USV Private Limited, Mumbai, IND

**Keywords:** glimepiride, metformin, fixed dose combination, long standing, durability

## Abstract

Objective

To evaluate the usage of various strengths of glimepiride and metformin fixed-dose combinations in the management of type 2 diabetes mellitus (T2DM) patients with comorbidities and complications.

Methods

A retrospective, non-randomized, non-comparative, multi-centric real-world study included T2DM patients (age > 18 years) taking glimepiride and metformin fixed-dose combinations. Age, duration of diabetes, diabetes complications, comorbidities (hypertension and dyslipidemia), dosage frequency, and concomitant medications were analyzed from medical charts.

Results

A total of 4858 T2DM patients were included, with a mean age of 52.67 years and males being predominant in the study population (60.85%). The laboratory investigations showed a mean glycated hemoglobin (HbA1c) of 7.5, low-density lipoprotein (LDL) cholesterol of 104.81 ± 38.19 mg/dL, and serum creatinine of 0.88 ± 0.26 mg/dL. Around 2055 (42.30%) T2DM patients were hypertensive, and telmisartan alone and a telmisartan-based combination were the drugs of choice for hypertension management. Similarly, 1073 (22.08%) T2DM patients were having dyslipidemia and were primarily managed with rosuvastatin and its combination in 664 (62%) patients. Macrovascular complications were observed in 339 (6.97%) T2DM patients, among which coronary artery disease (CAD) had maximum prevalence, affecting 273 (5.61%) T2DM patients. Microvascular complications were 1010 (20.79%) T2DM patients, among which neuropathy had affected a maximum of 686 (14.12%) followed by retinopathy (2.34%) and nephropathy (1.81%). Among the available 11 strengths, the glimepiride 2 mg and metformin 500 mg combination were most widely prescribed in 1297 (26.69%), followed by glimepiride 1 mg and metformin 500 mg in 1193 (24.57%) patients, and the preferred dosage pattern was twice a daily in 2665 (54.85%) T2DM patients. An age-wise prescription analysis showed that glimepiride and metformin combinations were the preferred choice for the management of diabetes across all the age groups.

Conclusion

The real-world evidence in the Indian clinical setting indicates that glimepiride and metformin fixed-dose combinations are widely used in the management in T2DM patients with comorbidities like hypertension, dyslipidemia, and diabetes complications. Glimepiride and metformin fixed-dose combinations are suitable for early as well as long-standing diabetes.

## Introduction

Diabetes mellitus has become a global burden, affecting 463 million people worldwide and responsible for 4.2 million deaths [1]. Type 2 diabetes mellitus (T2DM) is a heterogeneous disorder, and patients are at increased risk of developing macrovascular complications (coronary artery disease (CAD), peripheral arterial disease (PAD), and stroke/transient ischemic attack (TIA)) and microvascular complications (diabetic nephropathy, neuropathy, and retinopathy) due to inadequate glycemic control [2]. In India, the epidemic of diabetes continues to rise, which leads to disability and fatal complications that are related to an increased financial burden. According to the UK Prospective Diabetes (UKPDS) study, glycemic control using a single oral hypoglycemic agent is likely to be ineffective in the longer duration of disease, thus, unavoidably, many patients will require combination therapy to achieve their target glucose level [3]. The combination of modern sulfonylurea (glimepiride) and metformin is widely prescribed for effective blood glucose control due to its capability of counteracting “insulin secretion disorder” and “insulin resistance,” respectively. In India, multiple strengths of the glimepiride and metformin fixed-dose combination are available and widely used by primary care physicians and specialists [4]. The current study is aimed to analyze the usage of various strengths of the glimepiride and metformin combination in the management of T2DM patients with comorbidities and complications.

## Materials and methods

A retrospective, non-randomized, non-comparative, multi-centric, real-world study of Glycomet Glimepiride (REAL GGP) was conducted in Indian health care centers having the medical records of adult T2DM patients (age >18 years) who had received treatment with any strengths of glimepiride and metformin SR (sustained-release) combination as per the standard of care in a physician's practice. The data collected were analyzed for age, diabetes complications (microvascular and macrovascular), comorbidities (hypertension and dyslipidemia), the dosage frequency and strengths of the glimepiride and metformin combination, and the durability of glycemic control across all age groups (>18 years).

The study was conducted in accordance with the ethical principles that are consistent with the Declaration of Helsinki, International Conference on Harmonization-Good Clinical Practices, and the applicable legislation on non-interventional studies. The current study is Clinical Trials Registry India (CTRI) registered (CTRI/2018/09/015693) and the study protocol was approved by an Independent Ethics Committee.

## Results

Population characteristics

The study enrolled 4858 T2DM patients across India, i.e., 3030 patients from the south zone, 1239 patients from the east zone, 439 patients from the north zone, and 150 patients from the west zone. The mean age was 52.67± 11.53 years, with glycated hemoglobin (HbA1c) of 7.5%, 2956 (60.85%) patients were men, and mean weight was 71.07±11.59 kg. The laboratory investigations showed mean total cholesterol of 180.21 ± 42.36 mg/dL, low-density lipoprotein (LDL) cholesterol of 104.81 ± 38.19 mg/dL, high-density lipoprotein (HDL) cholesterol of 45.58 ± 18.34 mg/dL, triglyceride of 164.13 ± 81.74 mg/dL, and serum creatinine of 0.88 ± 0.26 mg/dL (Table 1).

**Table 1:** Baseline characteristic and laboratory investigations

Population Characteristics (n)	
Age (years, mean ±SD)	52.67 ± 11.53
Male, n (%)	2956 (60.85%)
Female, n (%)	1902 (39.15%)
Weight (kg, mean ±SD)	71.07±11.59
Laboratory Investigations	
HbA1c	7.5
FPG (mg/dL)	147.53 ± 48.99
PPG (mg/dL)	211.51 ± 64.99
Total Cholesterol (mg/dL)	180.21 ± 42.36
LDL (mg/dL)	104.81 ± 38.19
HDL (mg/dL)	45.58 ± 18.34
Triglycerides (mg/dL)	164.13 ± 81.74
Serum creatinine (mg/dL)	0.88 ± 0.26
Comorbidity	
Hypertension	2055 (42.30%)
Dyslipidemia	1073 (22.08%)
Macrovascular complications [n (%)]	
Coronary artery disease	276 (5.66%)
Stroke	63 (1.29%)
Microvascular complications [n (%)]	
Neuropathy	686 (14.12%)
Retinopathy	114 (2.34%)
Nephropathy	88 (1.81%)
Erectile dysfunction	85 (1.74%)
Foot ulcer	37 (0.76%)
Age-wise usage of glimepiride and metformin FDC [n (%)]	
18-40 years	696 (14.31%)
40-60 years	3798 (78.18%)
>60 years	365 (7.51%)

Clinical usage of glimepiride and metformin fixed-dose combinations

Strengths of the Combination

Among the available 11 strengths, the glimepiride 2 mg and metformin 500 mg combination were most widely prescribed in 1297 (26.69%) patients with T2DM, followed by glimepiride 1 mg and metformin 500 mg in 1193 (24.57%), and glimepiride 0.5 mg and metformin 500 mg in 640 (13.17%) patients with T2DM (Table 2).

**Table 2:** Usage of the various strengths of glimepiride and metformin combinations

Sr. No.	Strengths of glimepiride and metformin combinations	Number of patients (%)
1	Glimepiride 0.5 mg and Metformin 500 mg	640 (13.17%)
2	Glimepiride 0.5 mg and Metformin 1000 mg	175 (3.6%)
3	Glimepiride 1 mg and Metformin 500 mg	1194 (24.57%)
4	Glimepiride 1 mg and Metformin 850 mg	72 (1.48%)
5	Glimepiride 1 mg and Metformin 1000 mg	393 (8.08%)
6	Glimepiride 2 mg and Metformin 500 mg	1297 (26.69%)
7	Glimepiride 2 mg and Metformin 1000 mg	528 (10.86%)
8	Glimepiride 2 mg and Metformin 850 mg	232 (4.76%)
10	Glimepiride 3 mg and Metformin 1000 mg	200 (4.11%)
11	Glimepiride 4 mg and Metformin 1000 mg	127 (2.61%)

Dosage Pattern

**Figure 1:**
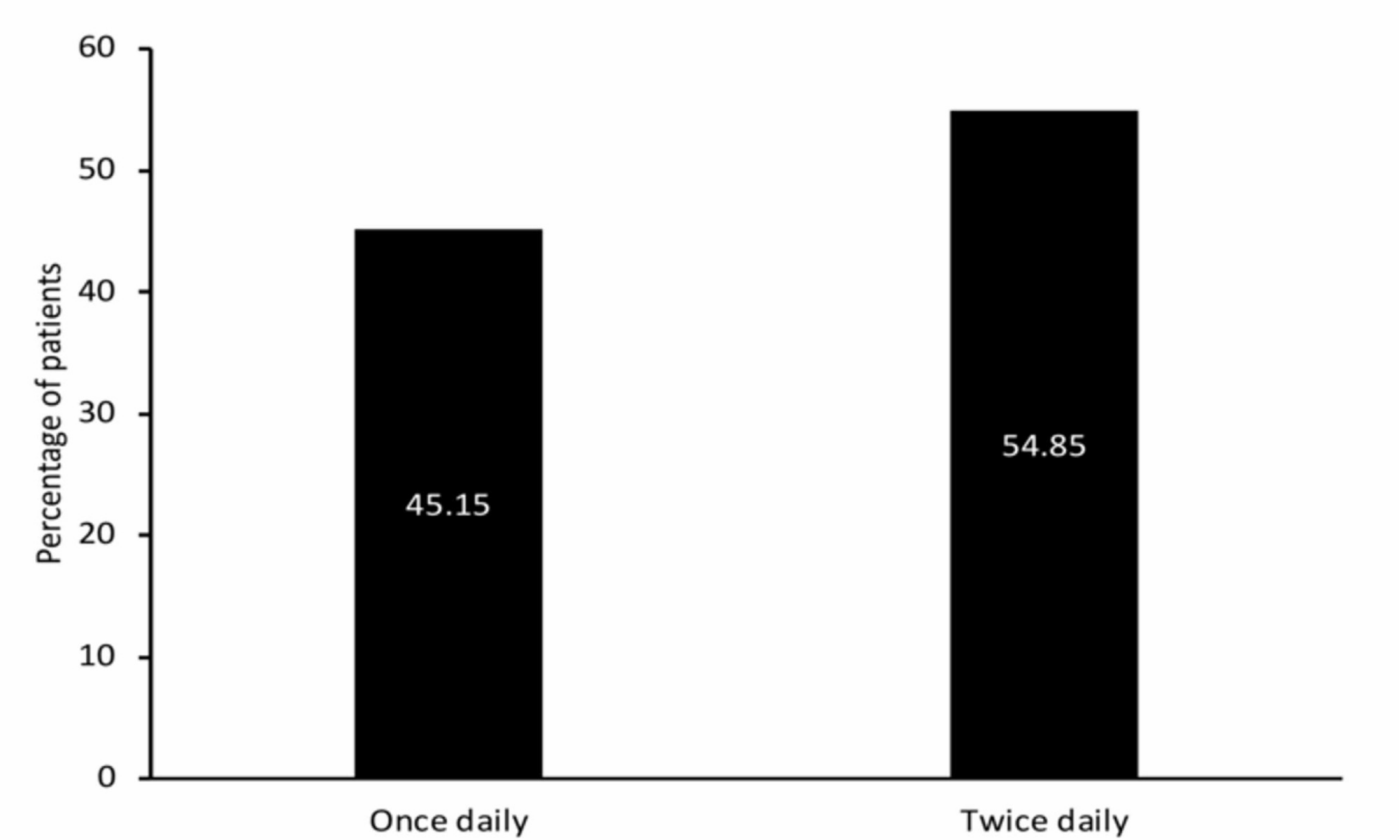
Dose frequency of glimepiride and metformin fixed-dose combinations

The prescription of glimepiride and metformin dosage was individualized according to the patients, among which BID (twice a day) was the preferred dosing frequency in 2665 (54.85%) patients with T2DM while OD (once a day) was prescribed in 2193 (45.14%) patients with T2DM (Figure 1).

Durability of the glimepiride and metformin combination

**Figure 2:**
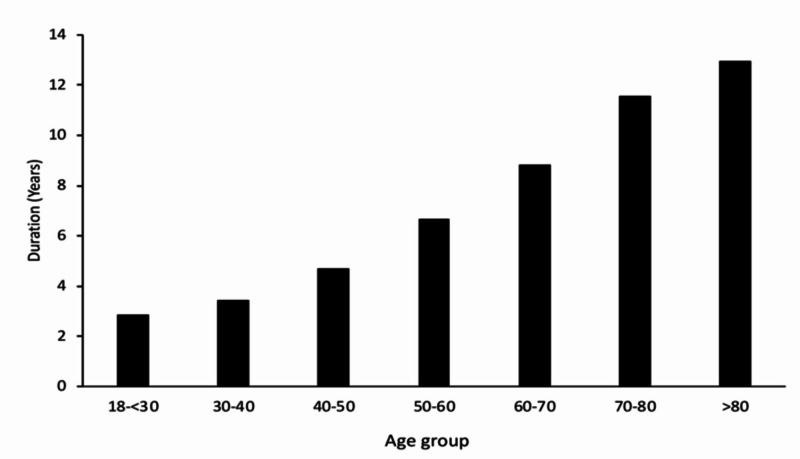
Duration of diabetes in patients taking glimepiride and metformin

Around 3798 patients with T2DM (78.18%) on the glimepiride and metformin combination were in the age group of 40-60 years, followed by 14.3% patients of <40 years of age, and 365 patients (7.15%) patients >60 years of age (Table 1). The duration of diabetes in the study population ranged from two years to 12 years across the age groups; this highlights the glycemic durability of the glimepiride and metformin combination (Figure 2). The mean duration of diabetes was 8.8 years, 11.5 years, and 12.9 years in patients of 60-70 years of age, 70-80 years of age, and over 80 years of age, respectively. All the strengths of glimepiride and metformin are used for T2DM patients >60 years of age and having long-standing diabetes.

Diabetes comorbidities

Hypertension

**Figure 3:**
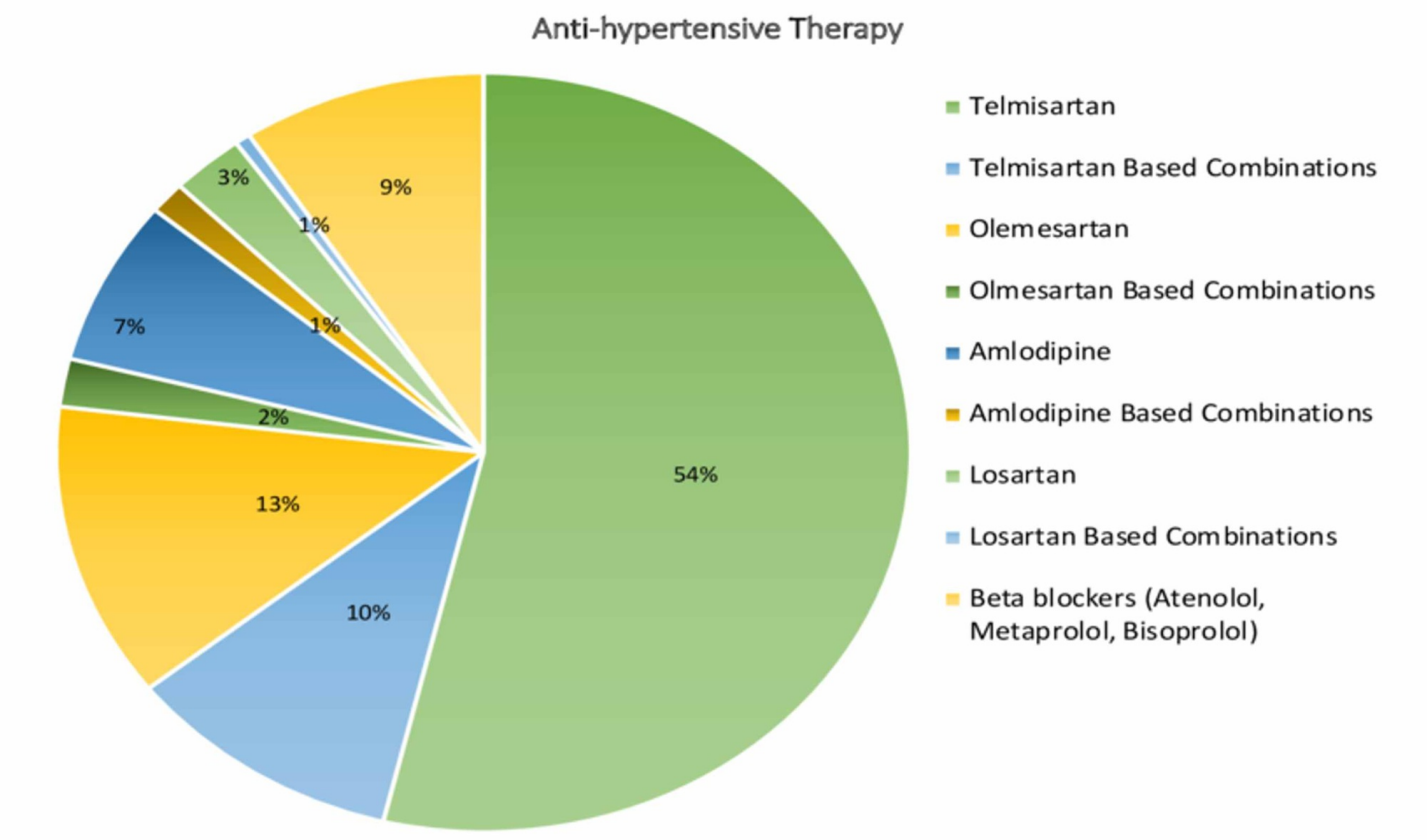
Anti-hypertensive therapy in hypertension with T2DM T2DM: type 2 diabetes mellitus

In total, 2055 (42.30%) patients with T2DM were having hypertension as comorbidity (Table 1). Telmisartan alone and telmisartan-based combinations were the most common antihypertensive therapy used in 1322 (64%) patients, followed by olmesartan and its combination in 301 (11%) patients (Figure 3).

Dyslipidemia

**Figure 4:**
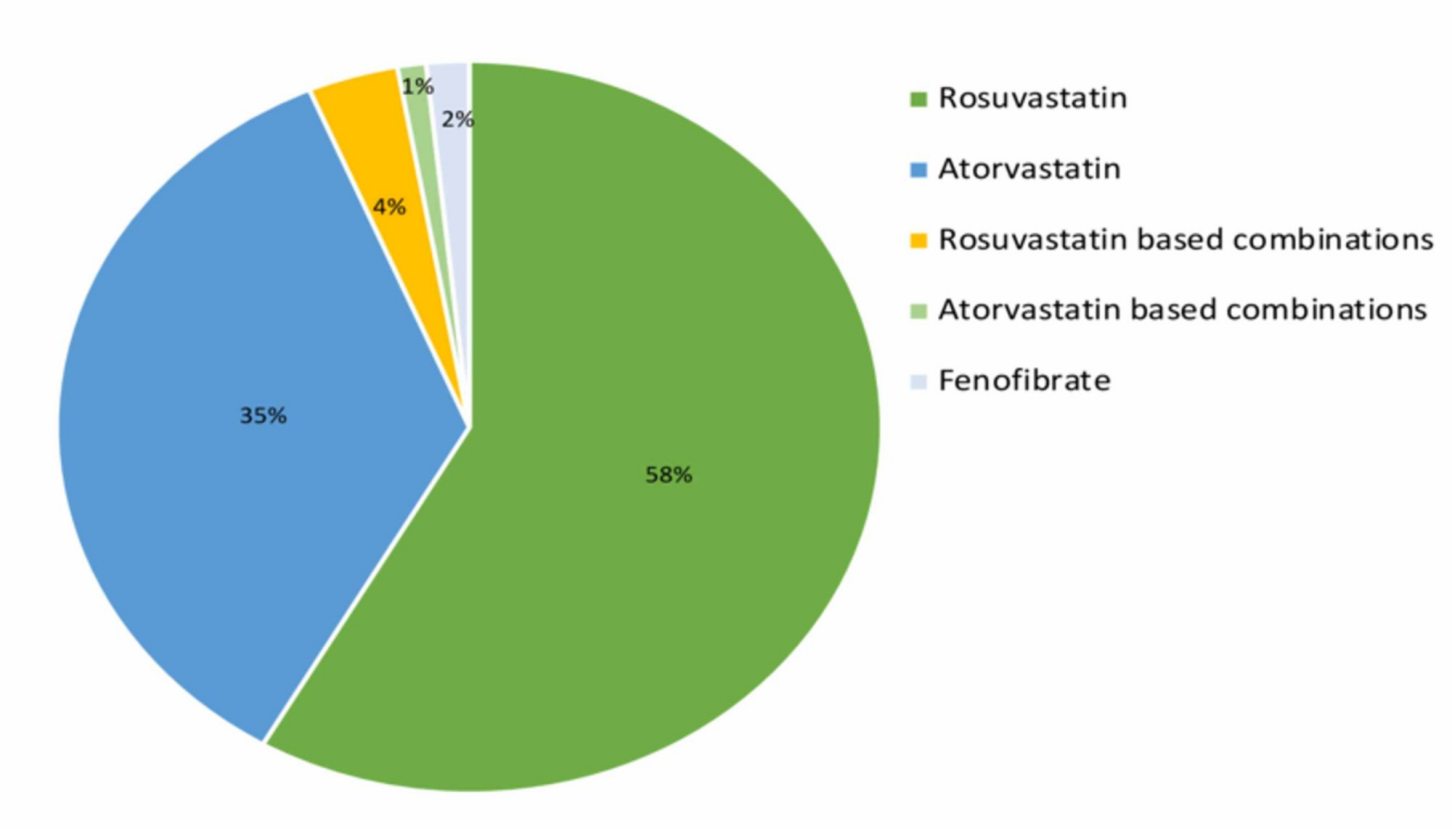
Lipid-lowering therapy in dyslipidemia with T2DM T2DM: type 2 diabetes mellitus

Dyslipidemia was observed in 1073 (22.08%) patients (Table 1). The study result showed that rosuvastatin and its combination was the choice of lipid-lowering therapy in 664 (62%) of patients, followed by atorvastatin and its combination in 391 (36%) patients with T2DM (Figure 4).

Diabetes complications

Macrovascular Complications

The results showed that 339 (6.97%) T2DM patients had macrovascular complications, among which coronary artery disease (CAD) was present in 276 (5.68%) T2DM patients, followed by stroke in 63 (1.29%) patients (Table 1). As primary or secondary prevention, aspirin was administered as monotherapy or dual antiplatelet therapy (DAPT) in ~7% of T2DM patients.

Microvascular Complications

Among the patients with T2DM, 1010 (20.79%) had microvascular complications. Neuropathy was the leading microvascular complication affecting 686 (14.12%) T2DM patients and the preferred choice of treatment was pregabalin and its combination. Retinopathy was observed in 114 (2.34%), nephropathy in 88 (1.81%), erectile dysfunction in 85 (1.74%), and foot ulcer in 37 (0.76%) T2DM patients.

## Discussion

All the strengths of the glimepiride and metformin combination are widely prescribed by the physicians across India, considering the efficacy and cost-effectiveness of the combination therapy [5].

The present real-world study describes the usage of various strengths of the glimepiride and metformin combination in patients with T2DM having hypertension and dyslipidemia as the major comorbidities and showing the presence of micro- and macrovascular complications.

The results of the present study indicate good glycemic control at all the age groups and showed a sustained legacy effect maintained for a longer duration of diabetes, which was in concordance with the study conducted by Kalra S et al. [4]. Early combination therapy with the glimepiride and metformin combination could provide the benefit of a legacy effect through earlier glucose control, avoiding a negative glycemic memory related to micro- and macrovascular complications [6]. Hypertension is a major risk factor for cardiovascular disease (CVD) [7], and the presence of hypertension in diabetes patients increases the risks of micro- and macrovascular complications and mortality [1].

In the present study, hypertension was the most common comorbidity observed, which highlights the fact that at least one antihypertensive drug should be administered to patients with diabetes to reduce cardiovascular (CV) morbidity and mortality [7]. As per Derosa G et al. antihyperglycemic drugs might have a small but clinically significant effect on blood pressure in patients with T2DM [8]. Despite the various anti-diabetic agents, the glimepiride and metformin combination was the preferred choice in hypertensive patients with diabetes for optimal blood glucose control, which was in concordance with the previous study by Tripathi S et al [5]. The hypertension treatment regimen in the current study showed that telmisartan and telmisartan-based combinations were the preferred choices of antihypertensive therapy in 64% of patients, followed by olmesartan and its combination in 11% of patients. These results were in concordance with Guerrero-García C et al. who stated that in hypertensive diabetes patients, multiple drug therapy is necessary to achieve blood pressure targets [9]. Studies report the CV protection of metformin, as it reduces the risk factors of CV diseases, including lipids, overweight, and hypertension; thus, lowering the incidence of CVD [10-11]. The conventional sulfonylureas are reported to have adverse CV effects while studies report glimepiride to have neutral CV effects [12]. A preliminary study of glimepiride conducted in 34 patients with T2DM showed positive effects on biomarkers related to CV regulation, indicating the presence of anti-oxidant, anti-inflammatory, and angiogenic properties [13].

Patients with T2DM have an increased prevalence of lipid abnormalities and, consequently, a high risk of atherosclerotic cardiovascular disease (ASCVD). Increased levels of LDL cholesterol and decreased levels of HDL cholesterol are the strongest predictors of CAD in patients with T2DM [14]. In the current study, dyslipidemia was observed in 22.08% of patients, which highlights the importance of lipid management as an integral part of T2DM management. Glimepiride does not show significant effects on lipid profile [15] while metformin is found to improve dyslipidemia in patients with T2DM by reducing LDL, triglycerides, and increasing HDL-C levels [16]. Kashi et al. [17] have reported a synergistic effect between metformin and statin, which is beneficial to reduce CV events in high-risk patients.

Macrovascular complications in diabetes primarily affect coronary arteries, peripheral arteries, and cerebrovasculature. Early macrovascular disease is associated with atherosclerotic plaque in the vasculature supplying blood to the heart, brain, limbs, and other organs while a late stage involves complete blockage of these vessels, which can increase the risks of myocardial infarction (MI), stroke, claudication, and gangrene [18]. Hence, the main target in the management of T2DM is maintaining good glycemic control, which is particularly important for controlling diabetes and preventing and delaying diabetes complications [19]. The CAROLINA (Cardiovascular Outcome Study of Linagliptin vs Glimepiride in Type 2 Diabetes) trial conducted in patients with T2DM at an elevated cardiovascular risk demonstrated that glimepiride is at par with linagliptin for safety from a three-point major cardiovascular event (3P-MACE) and 4P-MACE. Similarly, the South Asian Federation of Endocrine Societies (SAFES) consensus statement recommended that modern sulfonylureas (e.g. glimepiride) should be preferred over conventional sulfonylureas given the reduced mortality (all-cause and CV mortality) and better CV outcomes [20].

Micro-vessels are the basic functional unit of the CV system. They also regulate vascular permeability and myogenic responses, which can adapt blood flow according to local metabolic needs [18]. Diabetes induces pathognomonic changes in the microvasculature, affecting the capillary basement membrane, including arterioles in the glomeruli, retina, myocardium, skin, and muscle, by increasing their thickness, leading to the development of diabetic microangiopathy [21]. Hence, optimal glycemic control and achieving targeted blood glucose levels is important for the prevention of microvascular complications [22]. In the current study, 20.79% of patients with T2DM had microvascular complications, of which neuropathy was the leading microvascular complication affecting patients with T2DM and preferably treated with pregabalin and its combination. The UKPDS (United Kingdom Prospective Diabetes Study) showed that early intensive glucose control at the time of diagnosis is associated with a significant decrease in the risk of myocardial infarction and death from any cause, along with a reduction in the risk of microvascular disease [23], thus indicating the important role of glycemic control to reduce microvascular complications.

According to the American Diabetes Association (ADA) and the Research Society for Study of Diabetes in India (RSSDI) wheel, modern sulfonylureas, such as glimepiride have high efficacy over conventional sulfonylureas and lower risk of hypoglycemia with neutral CV risk [24-25].

A case-based questionnaire conducted with 2248 Indian patients showed that multiple strengths of glimepiride and metformin combinations are prescribed in T2DM patients, irrespective of age, diabetes duration, body mass index (BMI), and diabetes complications [26]. These combinations have shown good clinical efficacy, tolerability, and glycemic control and are beneficial particularly in resource constraint locations. The clinicians are benefited from these combinations due to the ease of up- and down-titration.

Glimepiride in combination with metformin is not only safe and effective but also cost-effective and an easily accessible approach for the management of T2DM [27]. A pharmacoeconomic study showed that the glimepiride and metformin combination was more cost-effective than the metformin and teneligliptin combination [28]. Glimepiride is comparable to other newer agents, such as DPP4i and GLP-1, in achieving the glycemic goal but at a low cost [29]; correspondingly, the results of reported studies confirm the cost-effectiveness of metformin over other anti-diabetic agents and lifestyle interventions [30]. These combinations are available widely in various strengths and easily accessible at affordable prices.

One of the key limitations of this study was the retrospective collection of data, which limited the strength of inference that can be made from the observations. The collection of data did not include fasting and the postprandial blood glucose levels and BMI of the patients.

## Conclusions

The real-world analysis concludes that all the strengths of the glimepiride and metformin combinations are widely prescribed in diabetes with comorbidities like hypertension and dyslipidemia and complications for optimal glycemic control. Glimepiride and metformin fixed-dose combinations are suitable for early as well as long-standing diabetes.

## References

[1] (2019). IDF diabetes atlas. 9th edition. https://diabetesatlas.org/en/.

[2] American Diabetes Association (2019). Microvascular complications and foot care: standards of medical care in diabetes—2019. Diabetes Care.

[3] Chawla A, Chawla R, Jaggi S (2016). Microvascular and macrovascular complications in diabetes mellitus: Distinct or continuum?. Indian J Endocr Metab.

[4] Kalra S, Das AK, Baruah MP (2019). Glucocrinology of modern sulfonylureas: Clinical evidence and practice-based opinion from an international expert group. Diabetes Ther.

[5] Tripathi S, Tiwaskar M, Kota S, Parthan G, Dasgupta A, Mohanasundaram S, Mohan V (2019). Need of single pill fixed-dose combination with glimepiride in management of diabetes mellitus. J Assoc Physicians India.

[6] Kim HS, Kim DM, Cha BS (2014). Efficacy of glimepiride/metformin fixed-dose combination vs metformin uptitration in type 2 diabetic patients inadequately controlled on low-dose metformin monotherapy: a randomized, open label, parallel group, multicenter study in Korea. J Diabetes Investig.

[7] Passarella P, Kiseleva TA, Valeeva FV, Gosmanov AR (2018). Hypertension management in diabetes: 2018 update. Diabetes Spectr.

[8] Derosa G, Sibilla S (2007). Optimizing combination treatment in the management of type 2 diabetes. Vasc Health Risk Manag.

[9] Guerrero-García C, Rubio-Guerra AF (2018). Combination therapy in the treatment of hypertension. Drugs Context.

[10] Loi H, Boal F, Tronchere H (2019). Metformin protects the heart against hypertrophic and apoptotic remodeling after myocardial infarction. Front Pharmacol.

[11] Charytan DM, Solomon SD, Ivanovich P (2019). Metformin use and cardiovascular events in patients with type 2 diabetes and chronic kidney disease. Diabetes Obes Metab.

[12] Geisen K, Végh A, Krause E, Papp JC (1996). Cardiovascular effects of conventional sulfonylureas and glimepiride. Horm Metab Res.

[13] Nakamura I, Oyama J, Komoda H (2014). Possible effects of glimepiride beyond glycemic control in patients with type 2 diabetes: a preliminary report. Cardiovasc Diabetol.

[14] American Diabetes Association (2019). Cardiovascular disease and risk management: standards of medical care in diabetes2125312019. Diabetes Care.

[15] Rosenblit PD (2016). Common medications used by patients with type 2 diabetes mellitus: what are their effects on the lipid profile?. Cardiovasc Diabetol.

[16] Lin SH, Cheng PC, Tu ST, Hsu​ SR, Cheng YC, Liu YH (2018;6). Effect of metformin monotherapy on serum lipid profile in statin-naïve individuals with newly diagnosed type 2 diabetes mellitus: a cohort study. PeerJ.

[17] Kashi Z, Mahrooz A, Kianmehr A, Alizadeh A (2016). The role of metformin response in lipid metabolism in patients with recent-onset type 2 diabetes: HbA1c level as a criterion for designating patients as responders or nonresponders to metformin. PLoS One.

[18] Rahelić D, Javor E, Lucijanić T, Skelin M (2017). Effects of antidiabetic drugs on the incidence of macrovascular complications and mortality in type 2 diabetes mellitus: a new perspective on sodium-glucose co-transporter 2 inhibitors. Ann Med.

[19] Kant R, Munir KM, Kaur A, Verma V (2019). Prevention of macrovascular complications in patients with type 2 diabetes mellitus: review of cardiovascular safety and efficacy of newer diabetes medications. World J Diabetes.

[20] Kalra S, Aamir AH, Raza A (2015). Place of sulfonylureas in the management of type 2 diabetes mellitus in South Asia: a consensus statement. Indian J Endocr Metab.

[21] Almourani R, Chinnakotla B, Patel R, Kurukulasuriya LR, Sowers J (2019). Diabetes and cardiovascular disease: an update. Curr Diab Rep.

[22] Aguiar C, Duarte R, Carvalho D (2019). Nova abordagem para o tratamento da diabetes: da glicemia à doença cardiovascular. New approach to diabetes care: from blood glucose to cardiovascular disease [Article in Portuguese, English]. Rev Port Cardiol.

[23] Stratton IM, Adler AI, Neil HA (2000). Association of glycaemia with macrovascular and microvascular complications of type 2 diabetes (UKPDS 35): prospective observational study. BMJ.

[24] Riddle MC (2019). A verdict for glimepiride: effective and not guilty of cardiovascular harm. Diabetes Care.

[25] Chawla R (2018). RSSDI Diabetes Update 2018. https://books.google.co.in/books/about/RSSDI_Diabetes_Update_2018.html?id=JkPowQEACAAJ&redir_esc=y.

[26] Unnikrishnan Ag, Pandit K, George J (2020). Clinical utilization pattern of multiple strengths of glimepiride and metformin fixed dose combinations in Indian type 2 diabetes patients. J Assoc Physicians India.

[27] Singh SK (2018). Commentary on "consensus recommendations on sulfonylurea and sulfonylurea combinations in the management of type 2 diabetes mellitus: International Task Force". Indian J Endocrinol Metab.

[28] Tandon T, Dubey AK, Srivastava S, Manocha S, Arora E, Hasan N (2019). A pharmacoeconomic analysis to compare cost-effectiveness of metformin plus teneligliptin with metformin plus glimepiride in patients of type-2 diabetes mellitus. J Family Med Prim Care.

[29] Zhang Y, McCoy RG, Mason JE, Smith SA, Shah ND, Denton BT (2014). Second-line agents for glycemic control for type 2 diabetes: are newer agents better?. Diabetes Care.

[30] Gu S, Tang Z, Shi L, Sawhney M, Hu H, Dong H (2015). Cost-minimization analysis of metformin and acarbose in treatment of type 2 diabetes. Value Health Reg Issues.

